# Nature-based cooling potential: a multi-type green infrastructure evaluation in Toronto, Ontario, Canada

**DOI:** 10.1007/s00484-021-02100-5

**Published:** 2021-03-30

**Authors:** Vidya Anderson, William A. Gough

**Affiliations:** 1 Climate Lab, University of Toronto Scarborough, Toronto, Canada; 2 Department of Physical and Environmental Sciences, University of Toronto Scarborough, Toronto, Canada

**Keywords:** Green roofs, Green walls, Urban agriculture, Urban forestry, Heat mitigation

## Abstract

The application of green infrastructure presents an opportunity to mitigate rising temperatures using a multi-faceted ecosystems-based approach. A controlled field study in Toronto, Ontario, Canada, evaluates the impact of nature-based solutions on near surface air temperature regulation focusing on different applications of green infrastructure. A field campaign was undertaken over the course of two summers to measure the impact of green roofs, green walls, urban vegetation and forestry systems, and urban agriculture systems on near surface air temperature. This study demonstrates that multiple types of green infrastructure applications are beneficial in regulating near surface air temperature and are not limited to specific treatments. Widespread usage of green infrastructure could be a viable strategy to cool cities and improve urban climate.

## Introduction

Urbanization and development transform the natural environment. As a result, local environmental stressors resulting from rising air temperatures are magnified (Revi et al. 2014). Climate change has an effect on the regional climate and the urban microclimate. The composition of the built environment has a significant impact on urban climate temperature regimes and the associated energy demands for heating and cooling (Revi et al. 2014; Jackson et al. 2010). Urban density, affluence, and energy consumption affect anthropogenic heat emissions and micro- and regional climate conditions (Revi et al. 2014, Jackson et al. 2010). Climate model projections have shown that tropospheric temperatures will continue to increase, and heat waves, frequency, and intensity will also increase (Hartmann et al. 2013). Regionally, heat waves are projected to increase in Ontario, Canada, consistent with rising tropospheric temperatures (Gough et al. 2016). Human activities are the main drivers in changing the Earth’s atmospheric and surface conditions, with significant global greenhouse gas emissions arising in urbanized areas (Lucon et al. 2014). Over half of the world’s population resides in urbanized areas, and by 2050, this is where most population growth is expected to occur (Lucon et al. 2014). Urbanized areas contribute approximately 75% of global carbon dioxide emissions, primarily generated by energy use (Revi et al. 2014). Sustainable approaches to urban development are necessary to cool cities, increase resilience, and improve urban climate.

As the climate evolves, extreme weather events, including heat waves, will continue to amplify in intensity, frequency, and duration, resulting in exacerbated human health risks (Seguin et al., 2008; Smith et al. 2014; Watts et al. 2015). Heat waves intensify pollen and aeroallergen levels triggering asthma, and these high air temperatures contribute to increasing levels of ozone and other air pollutants that affect cardiovascular and respiratory disease (PHAC 2018; WHO 2020). Heat stress and risks from food and waterborne illnesses will increase as a result of changing air temperatures (Smith et al. 2014; Watts et al. 2015; PHAC 2018). Warmer air temperatures have been linked to heat stroke, heat exhaustion, heat syncope, and heat cramps (Kovats and Hajat 2008).

Global and regional climate change projections were borne out when extreme heat events resulted in 70,000 additional deaths recorded across 12 countries in Europe in 2003, and the 2010 heat wave in Montreal resulted in approximately 300 additional deaths (Bustinza et al. 2013; Price et al. 2013). Approximately 120 heat-related deaths occur in Toronto annually, in addition to 120 and 40 deaths in Montreal and Ottawa, respectively (Cheng et al. 2005; Pengelly et al. 2007). Projections indicate that heat waves will continue to increase, and heat-related mortality is expected to double across Canadian cities including Toronto by 2050 and triple by 2080 (TPH 2005, 2013). Most recently, a heat wave swept Eastern Canada in the summer of 2018 with a death toll of 93 people in the province of Quebec (CTV News 2018).

Climate change mitigation has been defined as human intervention to reduce anthropogenic forcing of the climate system, including strategies to reduce net greenhouse gas emissions (Solomon et al. 2007; Anderson and Gough 2021a). On the other hand, climate change adaptation has been described as an adjustment in natural or human systems in response to actual or expected climatic stimuli or their effects, which moderates harm or exploits beneficial opportunities (Solomon et al. 2007; Anderson and Gough 2021a). The application of green infrastructure provides a nature-based solution that addresses both climate change mitigation and adaptation interventions and reduces the impact of atmospheric warming. Nature-based solutions have been defined by the International Union for Conservation of Nature (IUCN) as “actions to protect, sustainably manage, and restore natural or modified ecosystems, that address societal challenges effectively and adaptively, simultaneously providing human well-being and biodiversity benefits” (Cohen-Shacham et al. 2016). Nature-based solutions provide a framework for five categories of ecosystem-based approaches, one of which is green infrastructure (Cohen-Shacham et al. 2016; Cohen-Shacham et al. 2019; Seddon et al. 2020; Anderson and Gough 2021a).

## Literature review

For this study, the application of green infrastructure has been categorized into four areas: green roofs, green walls, urban vegetation and forestry, and urban agriculture systems (Anderson 2018; Anderson and Gough 2021a, b). Green infrastructure can reduce air and surface temperatures through shading and evapotranspiration which occurs when water moves from the earth to the atmosphere as it evaporates from the soil and other surfaces and from plant transpiration. Evaporation occurs with the movement of water from damp soil and vegetation. Transpiration occurs when water moves through plants along with nutrients. The combined process of evapotranspiration is energy driven and is amplified by temperature, radiation, and airflow. Temperature regulation varies with each type of green infrastructure application. It is influenced by a range of factors that include local climate, irrigation, physical dimension, vegetation, and seasonality.

The cooling benefits of green infrastructure have been well documented (Koc et al. 2018). Green infrastructure can cool the environment actively through evapotranspiration and passively through surface shading (Kleerekoper et al. 2012; Janhäll 2015; Nowak et al. 2006; Rao et al. 2014; King et al. 2014). Green infrastructure moderates temperatures, providing cooling capacity, and in urban settings reduces the urban heat island (Liang et al. 2014; Susca et al. 2011; Hall et al. 2012). Green infrastructure applications have demonstrated improved health outcomes from heat stress (Liang et al. 2014; Susca et al. 2011; Chen et al. 2014; Tzoulas et al. 2007; King et al. 2014; Nowak et al. 2006; Rao et al. 2014; Anderson 2018; Anderson and Gough 2020; Anderson and Gough 2021b).

Green infrastructure provides multiple benefits. These include the creation of green space, the mitigation of the urban heat island effect, cooling the environment, and the removal of air pollutants such as ozone and nitrogen dioxide (Alexandri and Jones 2008; Yang et al. 2008; Bowler et al. 2010; Baik et al. 2012; Gago et al. 2013; Berardi et al. 2014; Feng and Hewage 2014; Nowak et al. 2018; Sicard et al. 2018; Gourdji 2018; Anderson and Gough 2020). The application of green infrastructure has proven effective in reducing GHG emissions and reducing ambient carbon dioxide concentrations (Berardi et al. 2014; Alexandri and Jones 2008; Li et al., 2010; Marchi et al. 2014; Bowler et al. 2010; Hall et al. 2012; Velasco et al., 2016; Fargione et al. 2018; Graves et al. 2020; Anderson and Gough 2020). For example, green roofing and green wall technologies reduce air pollutant concentrations resulting in urban cooling (Speak et al. 2012; Kessler 2013; Anderson and Gough 2020). Other applications of green infrastructure such as urban vegetation strategies like tree and shrub plantings in urban corridors have also been shown to be effective in the reduction of temperatures (Nowak et al. 2006; Hall et al. 2012; Weber et al. 2014; Anderson 2018). Green roofs and urban vegetation can also function as an effective urban heat island mitigation strategy through their cooling effect on the urban microclimate (Wang et al. 2015; Berardi 2016; Jandaghian and Berardi 2019).

Various types of green infrastructure have the ability to regulate temperature. In the case of green roofs, various phenomena work together to regulate temperature. Foliage provides shading, enables thermal heat exchange, and absorbs thermal energy as part of photosynthesis, while soil and vegetation promote cooling through evaporation and transpiration (Berardi et al. 2014). Green roofs can also reflect up to 30% of solar radiation and absorb up to 60% through photosynthesis (Berardi et al. 2014). The application of a green roof reduces thermal loading (Li and Yeung 2014). A key factor in a green roof’s ability to regulate temperature is the abundance of vegetation (Weng et al. 2004). Maximizing surface area cover is integral although Morakinyo et al. (2017) argue that spatial coverage is less important than green roof type. For example, semi-intensive green roofs with higher leaf density and canopy height and 50% roof surface coverage showed a greater temperature reduction than extensive green roofs with 100% surface coverage. Thus, it should be acknowledged here that several types of green roofs exist and that spatial coverage is not the only key factor.

Green walls regulate temperature through shading, reducing reflected heat and evapotranspiration (Alexandri and Jones 2008; Demuzere et al. 2014; Elgizawy 2016). Air and surface temperature increases can be prevented by the application of green wall technology when significant quantities of solar radiation are transformed into latent heat as a result of evapotranspiration (Sheweka and Mohamed 2012). During the summer season, the application of a green wall can protect exterior walls from intense solar radiation and can both reflect and absorb up to 80% of radiation within its foliage (Sheweka and Mohamed 2012). The application of vegetation and foliage to building facades has been shown to decrease surface temperature (Hoelscher et al. 2016). Combining both green roof and green wall applications increases overall efficacy (Alexandri and Jones 2008).

Urban vegetation and forestry have been shown to be particularly effective in regulating air and surface temperatures. Trees provide shade cover that cools the air below (Bowler et al. 2010). Denser tree cover can provide further reductions in air and surface temperatures ((Bowler et al. 2010). Trees also reduce these temperatures through evapotranspiration. The cooling capacity of a single tree on a sunny day is equivalent to 20 to 30 kW (Kleerekoper et al. 2012).

The use of green infrastructure such as urban agriculture systems can regulate air and surface temperatures depending on ratio of vegetation and depth of soil or substrate (Lin et al. 2015). In addition to absorbing air pollutants and sequestering carbon, urban agriculture systems provide other ecosystem services (Thornbush 2015a, b; Lin et al. 2015; Thornbush 2015a, b). These services include the reduction of food miles and the carbon footprint associated with conventional agriculture. These can also reduce the pressures on conventional agriculture and thus improve food security when large-scale agricultural production is affected by weather extremes (Anderson 2018; Anderson and Gough 2020, 2021b).

Research on the temperature regulation benefits of green infrastructure has typically had a narrow focus on single applications and individual benefits (Alexandri and Jones 2008; Hall et al. 2012; Wang et al. 2015; Hoelscher et al. 2016). Research on the potential of surface temperature regulation has been limited to specific applications of green infrastructure; however, an evaluation across different applications simultaneously has not been undertaken.

**Fig. 1:**
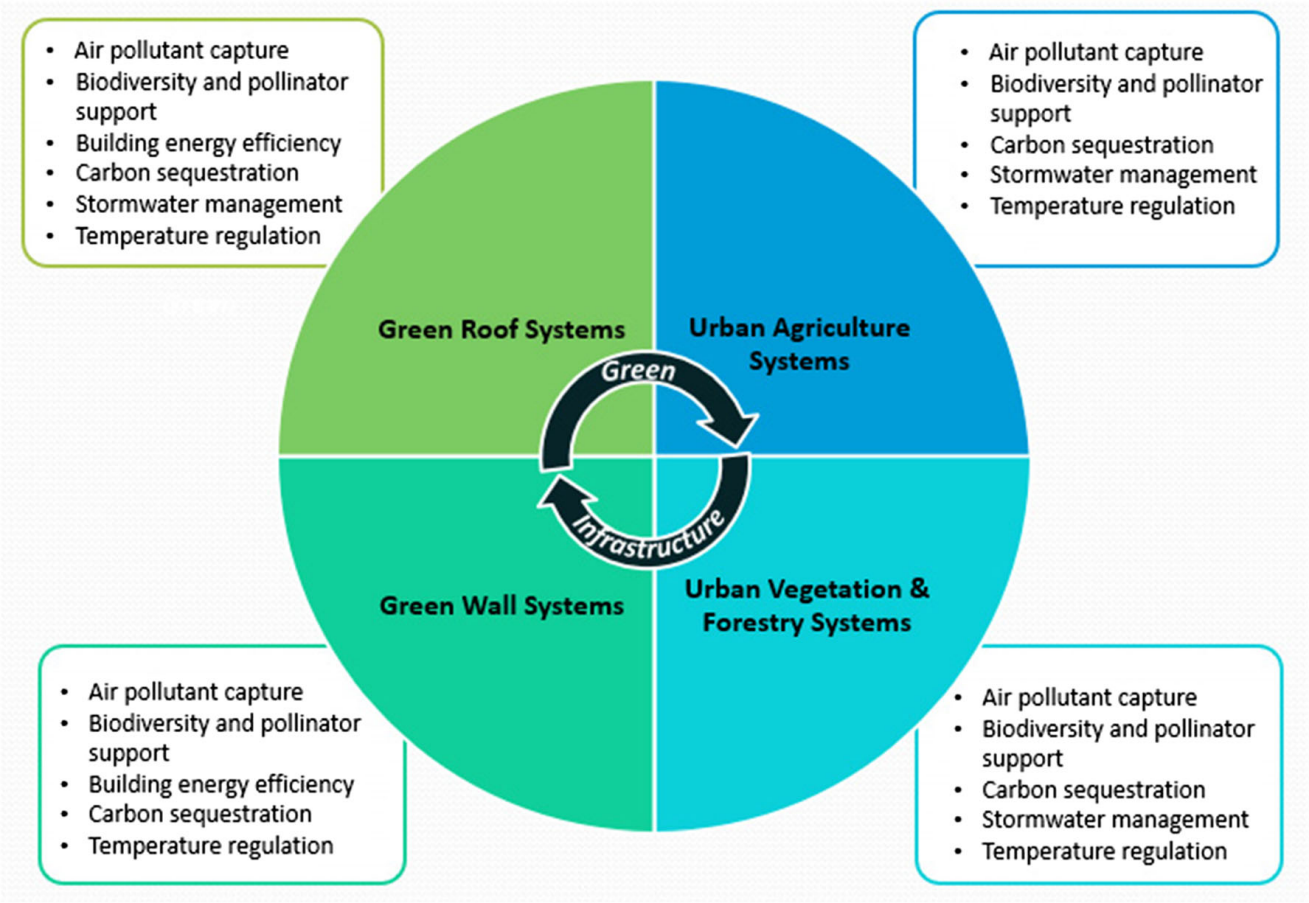
Green infrastructure form and function (Source: Anderson 2018; Anderson and Gough 2020; Anderson and Gough 2021a, b)

This study was undertaken to evaluate the potential of different green infrastructure applications to mitigate rising air and surface temperatures in Toronto, Ontario, Canada, by regulating surface temperature using consistent methodology across a range of treatments. Although the application of green infrastructure provides a mechanism for addressing climate change, each application is a complex climate change intervention with unique characteristics and multiple co-benefits that can be leveraged if strategically applied (Anderson and Gough 2020, 2021a, b). As illustrated in Fig. 1, there are some common functions shared among the green infrastructure applications. Other functions are exclusive to particular applications. The key function for this study is surface temperature regulation.

Green infrastructure is broadly defined as inter-connected networks of natural and engineered green spaces that provide a range of ecosystem services. As shown in Fig. 1 and noted above, applications of green infrastructure can be categorized into four areas: green roofs, green walls, urban vegetation and forestry, and urban agriculture systems (Anderson 2018; Anderson and Gough 2020, 2021a, b). Green roofs may be characterized as being extensive, weighing less as a result of shallower depth and also allowing for sloped roof application. Green roofs can also be characterized as being intensive wherein there is substantial depth to the soil layer and greater variety in vegetation (Berardi et al. 2014; Anderson 2018; Anderson and Gough 2020; Anderson and Gough 2021a, b). Green walls are building façades covered by plant growth or vegetated structures fixed to building facades fed by an automatic fertilization and hydration system (Voskamp and Van de Ven 2014; Marchi et al. 2014; Anderson 2018; Anderson and Gough 2020, 2021a, b). Urban vegetation and forestry include shrubs, bioswales (e.g. vegetated ditches for stormwater storage, drainage, and infiltration), green permeable pavements (e.g. paved surfaces replaced with grass or herbs), rain gardens, and trees (Nowak et al. 2006; Nowak et al. 2018; Voskamp and Van de Ven 2014; Anderson 2018; Anderson and Gough 2020; Anderson and Gough 2021a, b). Urban agriculture systems include growing roofs (i.e. food producing), rooftop gardens, market gardens, community gardens, and microgardens (Thornbush 2015a, 2015b; Lin et al. 2015; Anderson 2018; Anderson and Gough 2020, 2021a, b).

While there are clear benefits from the application of green infrastructure, there are associated costs with the design and construction of buildings and the conservation of natural areas. Although the upfront investment in green infrastructure can be more costly than conventional methods, the direct stormwater and wastewater management savings in avoided grey infrastructure installation, reduced sewage treatment costs, and avoided flood losses support the cost outlay. For example, the inclusion of green infrastructure within traditional stormwater asset portfolios can save up to 94% of life cycle costs (Berardi 2016; Jandaghian and Berardi 2019; Xu et al. 2019). In addition, a cost benefit analysis of street trees across five US cities realized an annual benefit of $1.50 USD to $3 USD for every dollar spent on tree management (GIO, 2020), while an analysis performed by the TD Bank found a benefit of $2.25 USD for every dollar spent on urban forestry in Toronto, Ontario, Canada (TD Economics 2014). There is no single application of green infrastructure suitable for all situations, since site, morphology, and budget are determining factors in choice. Green infrastructure investments are cost-effective and provide multiple economic benefits by improving property values and reducing stormwater management, air pollution, and electricity costs. For example, energy savings from green roofs range from 15 to 45% of annual energy consumption from reduced cooling costs (CCAP 2011; Demuzere et al. 2014; Cascone et al. 2018). Conservation of natural areas requires extensive planning and commitment to relinquish short-term development revenues; however, the ecosystem services provided compensate for the opportunity cost of development over the 50-year project life cycle (Nordman et al. 2018). The growth of street trees takes time before stormwater mitigation and other ecosystem services are fully established, but their benefits outweigh the lifetime costs (Nordman et al. 2018). Trees and vegetation can increase property values with increases ranging from 30 to 35% (CCAP 2011). The value of air pollution abatement by urban trees in the USA is estimated at $4B USD with the removal of 711,000 metric tonnes of pollutants annually (Nowak, 2006; Coutts and Hahn 2015), while trees in 86 Canadian cities removed 16,500 t of air pollution with human health effects valued at approximately $168 M USD (Nowak et al. 2018).

Building on the work undertaken by Anderson and Gough (2020), this study was undertaken to evaluate the potential of different green infrastructure applications to address rising temperatures in urban areas by regulating surface temperature. The objectives of this controlled field study were to evaluate the potential of multiple green infrastructure applications within the four categories shown in Fig. 1, to reduce near surface air temperature across different urban morphologies in Toronto, Ontario, Canada. The purpose of this study is not to evaluate the differences in green infrastructure performance between sites and morphologies. Rather, this study evaluates how different green infrastructure treatments can reduce near surface air temperature regardless of location, geography, or land use type.

## Methods

### Site selection

**Fig. 2:**
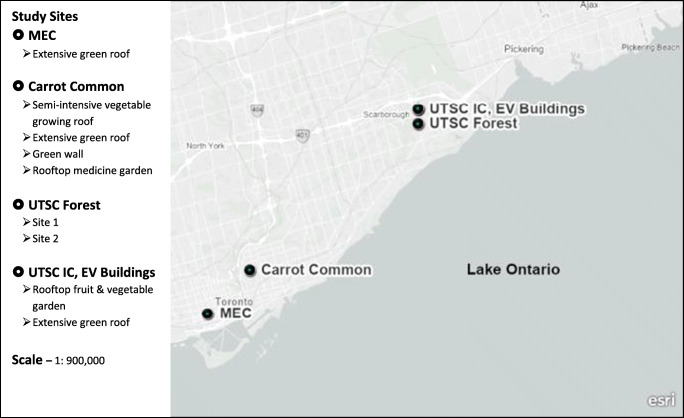
Map of study sites in Toronto, Ontario, Canada. MEC (Mountain Equipment Co-op), UTSC IC (University of Toronto Scarborough Instructional Centre), UTSC EV (University of Toronto Environmental Science and Chemistry Building)

The description of the study sites is provided in Anderson et al. (2018) and Anderson and Gough (2020). Part of that description is repeated here for the reader’s convenience. Field study sites were selected to be representative of the four green infrastructure categories and different urban morphologies, in addition to availability and accessibility of sites (Anderson 2018; Anderson and Gough 2020). A total of six sites were selected for the data collection campaign as shown in Fig. 2. Of these sites, three contained more than one application of green infrastructure. The six sites selected for the field study included: (1) the 186-m^2^ extensive green roof located at the Environmental Science and Chemistry building (EV) on the University of Toronto Scarborough (UTSC) campus in suburban Scarborough; (2) the 46-m^2^ rooftop fruit and vegetable garden located at the Instructional Centre (IC) building on the University of Toronto campus in suburban Scarborough; (3) two (2) urban forest sites located at the corner of Military Trail and Ellesmere Road on the University of Toronto campus in suburban Scarborough; (4) the 750-m^2^ multi-application site comprised of a herb and vegetable semi-intensive growing roof, an extensive green roof, a green wall, and a community rooftop medicine garden located at the Carrot Common in east Toronto; and (5) the 930-m^2^ extensive green roof located at the Mountain Equipment Co-op (MEC) outdoor retail store in downtown Toronto. As illustrated in Fig. 2, the MEC site is located in the downtown core of the city among a mix of tall high-rise buildings including residential condominium dwellings and commercial office towers. The Carrot Common site is located east of the downtown core in a highly urbanized area of mixed low-rise commercial and residential buildings. Each of the UTSC sites is located on the University of Toronto campus in Scarborough which is an eastern suburb of Toronto characterized by mixed residential, commercial, and industrial buildings that range from single-storey family dwellings to high-rise apartments and low-rise commercial and industrial building developments.

The City of Toronto has a continental climate moderated by its proximity to Lake Ontario and its southerly latitude. Toronto’s maximum air temperatures range from 23to 31 °C with moderate to high humidity. These are measured air temperatures at approximately 2 m above the surface, the standard for climatological data. Although Lake Ontario does have a cooling effect on the city, this can be very localized depending on wind speed and direction, and the lake effect can also increase the humidity and night-time minimum air temperatures. Air temperatures over 31 °C can occur during the summer where three consecutive days of such air temperatures are defined as an extreme heat event in southern Ontario (Anderson 2016). Coupled with high humidity, the humidex values can climb into the 40 °C range during a summer heat event (Gough and Rosanov 2001; Gough et al. 2002; Mohsin and Gough 2010; Gough and Sokappadu 2016; Anderson et al. 2018).

### Field study

A data collection campaign was undertaken across different applications of green infrastructure including green roofs, green walls, urban vegetation and forestry, and urban agriculture systems (Fig. 1). This data collection campaign to measure the potential of green infrastructure to regulate near surface air temperature was undertaken using climate monitors at fixed locations over the course of two summer seasons from July through August 2016 and July through August 2017. To provide some context and to expand on the general climate conditions described in the previous section, the climate normals for Toronto, Ontario, Canada (1981–2010), for the months of July and August for the mean daily air temperature are 22.3 °C and 21.5 °C, respectively. For the summer of 2016, the July and August air temperatures were 23.8 °C and 24.3 °C, respectively, warmer than the climate normals by two standard deviations. The summer of 2016 was the warmest in Toronto, Ontario, Canada, in over 15 years. The summer of 2017 was cooler with 21.8 °C and 20.6 °C for July and August. These values were cooler than the climate normals by less than one standard deviation.

### Monitoring schedule and sensor calibration

During the data collection campaign to measure the potential of green infrastructure to regulate near surface air temperature, monitoring was undertaken on a continuous basis for July and August 2016 and July and August 2017 (Anderson 2018). At each site, data loggers were installed to continuously measure and archive hourly near surface air temperatures. Hourly measurements are a standard sampling period in climatic studies (Gough et al. 2020). The temperature loggers were tested and calibrated during the spring season of 2016, and periodic checks were conducted through 2016 and 2017 to check battery life and functionality of the loggers and to download data. Calibration was undertaken by assembling all the data loggers together to synchronize the time and to verify that each data logger was reading the same values simultaneously.

### Monitoring

**Fig. 3:**
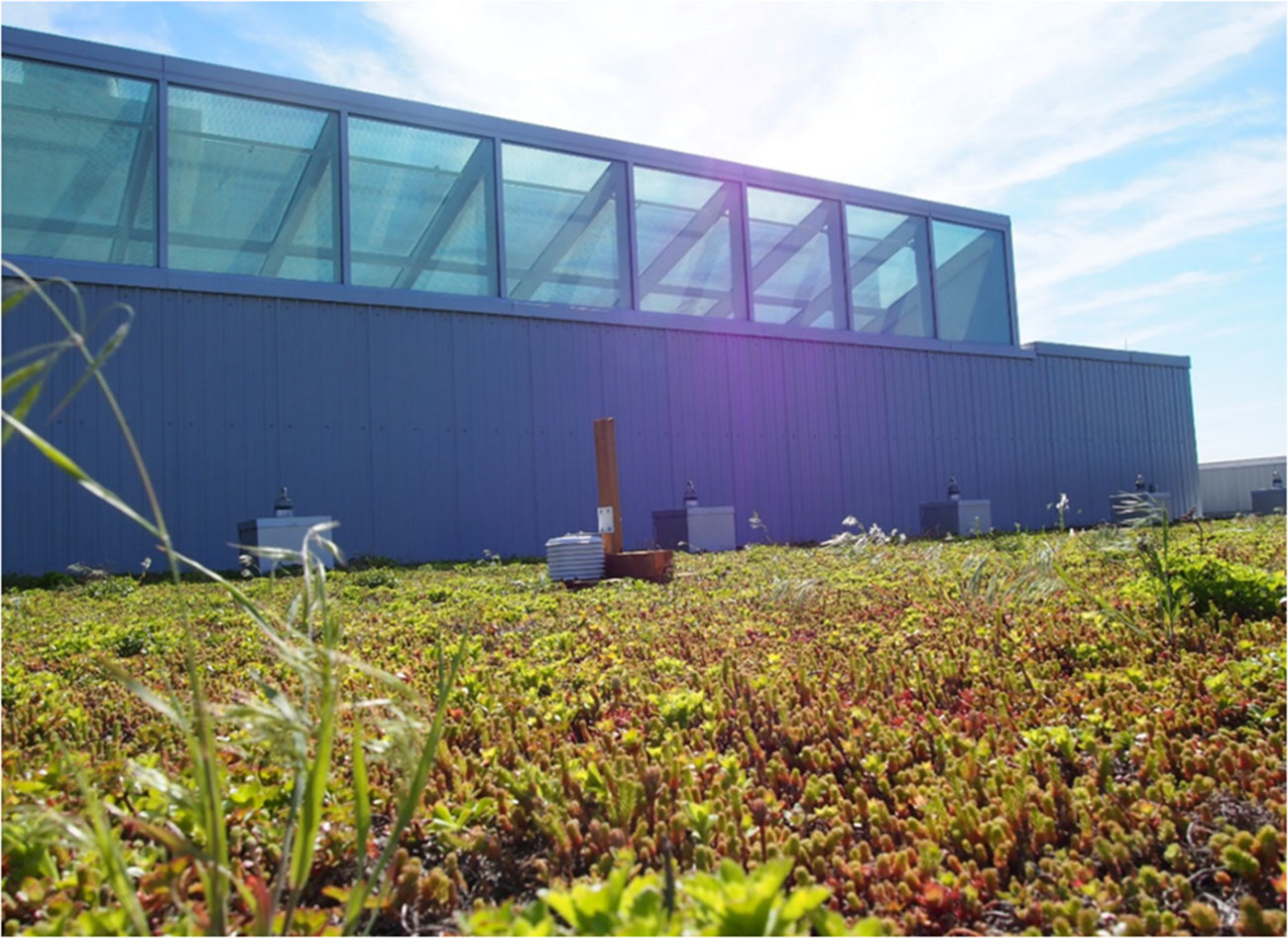
HOBO monitor installation on UTSC EV green roof

During the data collection campaign, two locations were set up at each site to establish a control and a treatment position for the temperature loggers (Anderson 2018). Control and treatment positions for each site were established approximately 30 to 50 m apart to minimize any influence on the control positions from the treatment areas. One logger each was positioned directly on the control or green infrastructure application surface being monitored to capture the flow of air across the logger. The loggers were housed within weatherproof shields (i.e. Stevenson screens) and mounted on wooden stands for stabilization. The wooden stands were installed at a height of approximately 2.5 cm above the control and green infrastructure treatment surfaces as shown in Fig. 3 to minimize the influence of larger scale wind flows on the near surface air temperature and to avoid epiclimatic variations at the surface. The data loggers installed for this purpose were the Onset HOBO weatherproof temperature loggers (model U23) which are designed to collect temperature data in outdoor environments. Detection limits for the HOBO model U23 weatherproof temperature loggers range from −40 °C at its coldest limit to 70 °C at its warmest limit of operational temperatures. The HOBO model U23 temperature logger is a high precision instrument with accuracy of ±0.21 °C from 0° to 50 °C (± 0.38 °F from 32° to 122 °F). The data collected from treatment and control sites were compared using a Student’s *t* test as was done in Anderson and Gough (2020).

## Results

The data collected was used to evaluate the potential impact of green infrastructure to regulate near surface air temperature.

### Temperature regulation

The data collected show that there was a reduction in near surface air temperature between the treatment and control applications across all sites during the summer months of July and August 2016 and July and August 2017 as shown in Tables 1 and 2. The reduction played out differently for different green infrastructure and locations. For the maximum temperature of the day (typically occurring mid-afternoon), the temperature reduction for all but two locations is substantial exceeding on average 0.6 °C (Table 1). The two exceptions occurred at the Carrot Growing Roof and Carrot Medicinal Garden. Two factors appear to be at play for these, the nature of the green infrastructure treatment and the location of an air conditioner outlet vent. The Carrot Medicinal Garden and the Carrot Growing Roof were green infrastructure treatments that provide less dense coverage than the Carrot Green Roof and Carrot Green Wall. As well, both treatments were located in close proximity to an air conditioning vent. For the minimum temperature of the day (Table 2), the reduction is muted for most locations and treatments compared to the daytime reduction. The two exceptions are the Carrot Medicinal Garden and Carrot Growing Roof. These are well watered green infrastructure treatments that are likely cooled by nighttime evapotranspiration and not mitigated by the air conditioning venting at night.

**Table 1:** The impact of green infrastructure on the average minimum daily temperature for July-August 2016

**Location**	**Control**		**GI**		**Delta**	
	*Tmax*	STD	*Tmax*	STD	*Tmax*	STD
MEC Green Roof	29.48	3.51	27.88	3.30	1.60	0.88
Carrot Green Roof	30.33	3.47	28.37	2.88	1.96	1.16
Carrot Growing Roof	30.33	3.47	30.25	3.34	0.08	1.13
Carrot Medicinal Garden	30.33	3.47	30.61	3.29	− 0.29	1.24
Carrot Green Wall	30.33	3.47	28.45	3.40	1.87	0.93
UTSC EV Green Roof	28.43	3.81	27.68	3.35	0.75	1.82
UTSC IC Roof Top Garden	28.62	3.20	27.14	3.01	1.49	1.17
UTSC Forest Residence	27.04	3.43	26.41	3.20	0.63	0.63
UTSC Forest	27.04	3.43	26.11	3.23	0.93	0.75

Reported are control and treatment (GI) temperatures, their difference (delta), and their standard deviations (STD all in °C

**Table 2:** The impact of green infrastructure on the average minimum daily temperature for July-August 2017

**Location**	**Control**		**GI**		**Delta**		
	*Tmax*	STD	*Tmin*	STD	*Tmin*	STD	*Mean*
MEC Green Roof	19.62	2.46	19.41	2.46	0.21	0.19	0.90
Carrot Green Roof	17.88	2.88	16.99	3.30	0.88	0.70	1.42
Carrot Growing Roof	17.88	2.88	16.58	3.36	1.29	0.86	0.69
Carrot Medicinal Garden	17.88	2.88	16.29	3.20	1.59	0.79	0.65
Carrot Common Green Wall	17.88	2.88	18.08	2.81	−0.20	0.25	0.84
UTSC EV Green Roof	18.06	2.58	17.36	2.75	0.70	0.47	0.73
UTSC IC Roof Top Garden	17.73	2.69	17.42	2.66	0.31	0.45	0.90
UTSC Forest Residence	17.94	2.70	17.88	2.64	0.07	0.19	0.35
UTSC Forest	17.94	2.70	17.81	2.56	0.14	0.35	0.53

Reported are control and treatment (GI) temperatures, their difference delta), and their standard deviations (STD all in °C. In the final column the average of the average minimum and maximum temperature is reported as the average mean daily temperature (mean).

Of the green infrastructure applications tested, urban agriculture systems showed the greatest impact in regulating near surface air temperature with an observed monthly average reduction in temperature. The application of green roof systems shows an average reduction in near surface air temperature of 0.8 °C (Table 2) with an observed average monthly reduction as high 1.4 °C on the Carrot Green Roof. Urban forestry and vegetation systems showed an average reduction in near surface air temperature of 0.44 °C at the two UTSC forest sites. Green wall systems show an average reduction in near surface air temperature of 0.8 °C on the Carrot Green Wall (Table 2).

### Analysis of near surface air temperature regulation

**Fig. 4:**
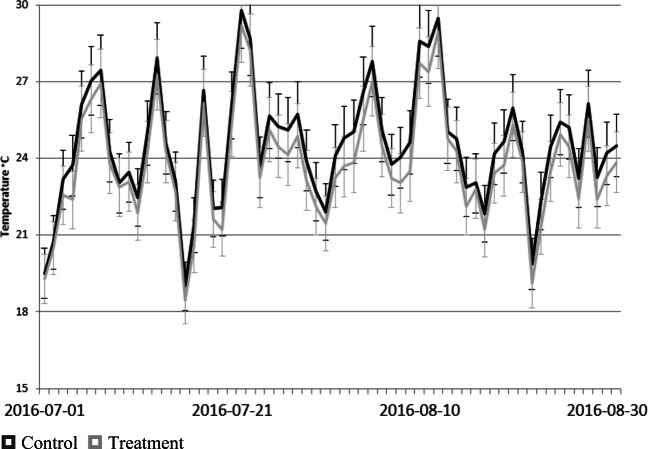
Daily average near surface air temperature **(°C)** for control and treatment across all sites for the 2016 summer season. The error bars represent 5% of the measured value, consistent with the detection limits of the instrumentation.

**Fig. 5:**
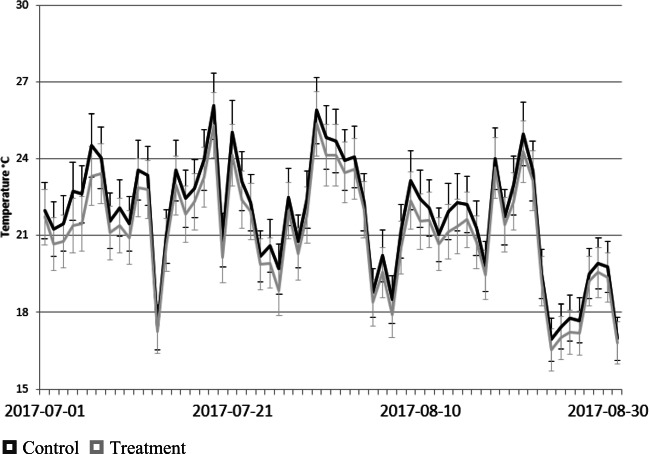
Daily average near surface air temperature (°C) for control and treatment across all sites for the 2017 summer season. The error bars represent 5% of the measured value, consistent with the detection limits of the instrumentation.

The daily average of the near surface air temperature treatment values is less than the control values for most dates when monitoring took place during the 2016 and 2017 summer seasons as shown in Figs. 4 and 5.

A *t* test was conducted for the treatment and control pair at each site, and all had a *p* value below the 0.001 margin of error, showing that there is a statistically significant difference in near surface air temperature between the means of the treatment and control for all applications of green infrastructure. This confirms the observed reduction in near surface air temperature between the treatment and control applications.

**Fig. 6:**
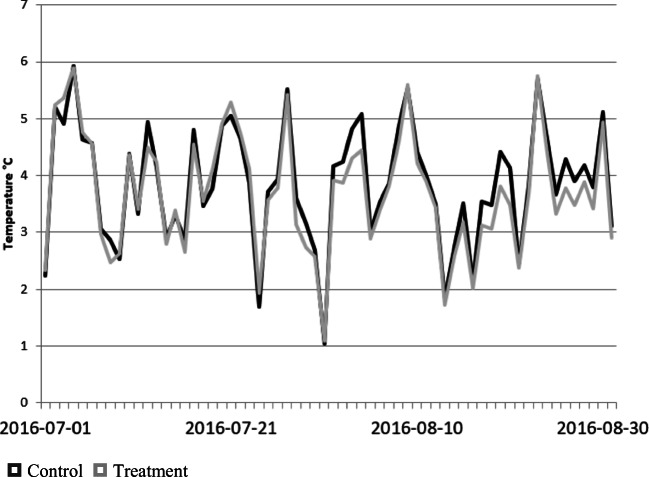
Daily near surface air temperature standard deviation for control and treatment across all sites for the 2016 summer season

**Fig. 7:**
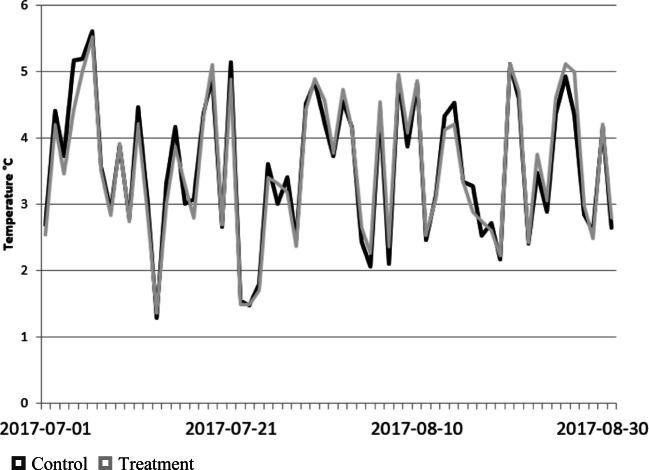
Daily near surface air temperature standard deviation for control and treatment across all sites for the 2017 summer season

The standard deviation for the treatment and control sites are presented in Figs. 6 and 7 for the two summers illustrating almost identical behaviour for the two. This suggests that the green infrastructure treatments while lowering the measured near surface air temperature do not change its variability.

## Discussion

Analysis of the data collected to measure the potential of green infrastructure to regulate near surface air temperature is consistent with the hypothesis that multiple types of green infrastructure have the potential to mitigate rising near surface air temperatures in Toronto, Ontario, Canada, regardless of location, geography, or land use type.

Observations from this controlled field study confirm that green infrastructure has a positive, statistically significant impact on near surface air temperature reduction. The findings of this study indicate that the application of green infrastructure across different urban morphologies in Toronto, Ontario, Canada, is beneficial in mitigating rising near surface air temperatures. Observations from this controlled field experiment show a maximum average monthly near surface air temperature reduction that ranged between 0.3 and 1.3 °C. The potential of green infrastructure writ large to mitigate local environmental stressors such as heat waves resulting from higher temperatures is significant. These results are consistent with previous work (Alexandri and Jones 2008; Hall et al. 2012; Wang et al. 2015; Hoelscher et al. 2015; Susca et al. 2011; Liang et al. 2014), which has focused primarily on single applications of green infrastructure. Our results complement other work undertaken in Toronto, Ontario, Canada, that evaluates the impact of different treatments including specific applications of green infrastructure (i.e. green roofs and urban forestry and vegetation) on urban microclimate and temperature regulation. These studies were undertaken using microclimate simulations that vary roof surfaces, pavement material, building height, and new construction (Wang et al. 2015; Berardi and Wang 2016; Berardi 2016; Taleghani and Berardi 2018; Jandaghian and Berardi 2019).

Compared to these studies, this field study is novel because multiple types of green infrastructure were tested simultaneously in a controlled field experiment using consistent methodology across a range of treatments in Toronto, Ontario, Canada. Observations indicate that different green infrastructure applications are beneficial in regulating near surface air temperature and are not limited to single or specific applications such as green roofs, green walls, or trees. In addition, this research shows the potential temperature regulation benefits of productive green infrastructure applications (e.g. urban agriculture systems) that have not been previously evaluated in other studies. This study indicates that urban agriculture systems have the potential to reduce near surface air temperatures in urban and suburban settings with the additional co-benefit of enhanced food security. Given that the built environment has a significant impact on urban climate temperature regimes and the associated energy demands for heating and cooling, the wide adoption of multiple green infrastructure applications within Toronto, Ontario, Canada, could be meaningful in mitigating rising temperatures in urbanized areas using nature-based solutions.

A number of studies show that there are differences in cooling effect across different applications of green infrastructure. For example, urban trees are more effective in reducing outdoor temperatures than green walls and green roofs (Perini and Magliocco 2014; Zolch et al. 2016; Erell 2017). Although trees have demonstrated greater efficacy in reducing outdoor temperatures, combining green infrastructure applications like green roofs and green walls can increase overall efficacy in addition to requiring less space than trees alone (Alexandri and Jones 2008; Jayasooriya et al. 2017). It should be noted that configuration, orientation, and building materials can have a significant impact on cooling magnitudes; however, the experimental design of this study includes closely located control and treatment positions, controlling for local environmental conditions and building materials. These factors may impact the absolute value of temperatures but not the difference between the treatment and control of the paired experiment. Of the green infrastructure applications tested in this study, urban agriculture systems showed the greatest impact in regulating near surface air temperature with an observed monthly average reduction in near surface air temperature. The urban agriculture systems tested in this study included a semi-intensive growing roof and two rooftop gardens. This supports the findings of Morakinyo et al. (2017) that spatial coverage is less important than green roof type. For example, semi-intensive green roofs with higher leaf density and canopy height and 50% roof surface coverage showed a greater temperature reduction than extensive green roofs with 100% surface coverage.

We note two other limitations of this study. The first is spatial sampling, and the second is temporal sampling. For each site, there was one data logger each for the control and experimental sites. To control potential spatial variation within a location or the potential of edge effects, more spatial sampling is required to ensure the ubiquity of the results. The sampling was done over two summers. While the similarity of the results for the two summers was reassuring from an experimental design perspective, more years of monitoring would confirm the results obtained in this study. Moving forward two other aspects are of interest. The first is the spatial extent of the impact of the green infrastructure treatment, both vertical as well as horizontal, that has downstream effects. The second is to explore the seasonality of the green infrastructure treatment. The experiment focused on the summer months of July and August. It is of interest to explore the impact of green infrastructure treatments during the growing period in the spring and the senescence period in the autumn.

## Conclusions

People suffer illnesses and experience reduced quality of life when high temperatures occur for an extended period of time. High temperatures contribute directly to deaths from cardiovascular and respiratory disease, particularly among the elderly, and individuals who are chronically ill and socially disadvantaged are more vulnerable to the health effects of extreme heat (Smith et al. 2014; Watts et al. 2015; PHAC 2018). Rising temperatures as a result of climate change will continue to intensify these problems, aggravating the burden of illness (Smith et al. 2014; Watts et al. 2015; WHO, 2018). Urbanization, social disparity, and an ageing population will further exacerbate the health impacts of rising temperatures. Sustainable approaches to urban development are necessary to cool cities, increase resilience, and improve urban climate.

The results of this field study, while limited to two summer seasons as noted above, provide insight into the impact of multiple green infrastructure applications (e.g. green roof systems, green wall systems, urban agriculture systems, and urban forestry and vegetation systems) on surface temperature regulation in Toronto, Ontario, Canada. Of the green infrastructure applications tested, urban agriculture systems showed the greatest impact in reducing temperature. The application of green roof systems showed an average reduction in near surface air temperature of 0.5 °C with an observed average monthly reduction as large as 0.9 °C. Urban forestry and vegetation systems showed an average reduction in temperature of 0.44 °C with an observed average monthly reduction as large as 0.6 °C. Green wall systems showed an average reduction in temperature of 0.5 °C with an observed average monthly reduction as large as 0.6 °C. Although reductions in temperature vary across applications of green infrastructure, there is significant potential to address rising temperatures by optimizing the built environment to not only regulate temperature and improve the urban microclimate but to leverage other co-benefits such as stormwater matter management; air pollution abatement; biodiversity and pollinator support; and enhanced food security through the application of productive green infrastructure such as growing roofs and rooftop gardens. This study shows that multiple types of green infrastructure applications are beneficial in regulating temperature and are not limited to single or specific applications. Green infrastructure offers a multi-faceted, nature-based solution to the challenges presented by different urban morphologies in a changing climate.
